# Is tumour location a dominant risk factor of recurrence in early rectal cancer?

**DOI:** 10.1007/s00464-024-11413-6

**Published:** 2024-12-16

**Authors:** Roberto Rosén, Henrik Thorlacius, Carl-Fredrik Rönnow

**Affiliations:** Department of Clinical Sciences, Division of Surgery, Skåne University Hospital, Lund University, 20502 Malmö, Sweden

**Keywords:** Rectal cancer, Early rectal cancer, Lymph node metastasis, Cancer recurrence, Tumour location, Risk factors

## Abstract

**Background:**

Impact of rectal tumour location on risk of lymph node metastases (LNM) and recurrence in early RC is poorly studied and elusive. Tumour location as a prognostic factor may contribute to optimise management of early RC in the future. The aim of this study was to investigate rectal tumour location as an independent predictor of oncologic outcome in early rectal cancer (RC).

**Methods:**

Retrospective multicentre national cohort study on prospectively collected data on all patients with T1-T2 RC, undergoing surgical resection between 2009 and 2021. Tumour location was categorised as distal (0–5 cm), mid (5–10 cm), and proximal (10–16 cm), measured from the anal verge.

**Results:**

Incidence of LNM in the 2424 included T1–T2 RC patients was 18.2%, 17.3% and 21.6% for distal, mid and proximal tumours, respectively. Recurrence was detected in 130 (7.6%) out of 1705 patients available for recurrence analyses (60-month median follow-up). Incidence of recurrence was twice as high in distal (11.4%) compared to proximal (5.6%) tumours and was 8.3% in mid located tumours. Distal (HR 2.051, CI 1.248–3.371, *P* < 0.05) and mid (HR 1.592, CI 1.061–2.388, *P* < 0.05) tumour location were significant risk factors of recurrence in uni- and multivariate Cox regression analyses.

**Conclusions:**

This study shows that tumour location significantly affects incidence of recurrence in early RC, with an increasing risk for mid and especially distal location, found to be a predominant risk factor of recurrence. Our findings stress the need for an increased awareness on differences in oncologic outcome related to tumour location in early RC.

**Supplementary Information:**

The online version contains supplementary material available at 10.1007/s00464-024-11413-6.

Risk stratification is pivotal in modern management of rectal cancer (RC), allocating patients to the most beneficial treatment alternative. Local resection has become a feasible and desirable treatment option in selected cases of early-stage RC (pT1-2), avoiding the heavy burden of surgical resection with risk of complications and functional impairment. However, risk of leaving lymph node metastases (LNM) untreated, as well as risk of recurrence limits local resection as final treatment to low-risk T1 RC [1]. Consequently, high-risk T1 and all T2 tumours are recommended surgical resection, although local resection can sometimes be an option in patients deemed unfit for surgery or combined with adjuvant chemo-/radiation therapy, within trials [2]. However, the literature on risk factors predicting LNM as well as recurrence in early RC is heterogenous and contradictive [3–7]. In addition, almost 70% of T1 RC are classified, by current guidelines as high-risk of LNM but the actual incidence of LNM is reported to range from 10 to 18% [7–10], making surgical resection an overtreatment in the majority of cases. In this context, it is interesting to note that a growing body of evidence suggest that tumour biology and genomics in colorectal cancer differs greatly depending on tumour location, affecting oncologic outcome and prognosis. For example, previous studies have shown that risk of recurrence is higher in rectal compared to colon cancer and higher in right-sided compared to left-sided colon cancer [11–13]. Some studies have even suggested that distal RC have a higher risk of recurrence compared to mid/proximal RC [14–17], which lies in contrast to other studies, reporting no difference in oncologic outcome based on tumour location [5, 10, 18–21]. Thus, the importance and impact of rectal tumour location on oncologic outcome remains elusive and is poorly studied in early RC specifically. It is also interesting to note that the majority of studies investigating risk factors of LNM and recurrence, as well as studies comparing local and surgical resection, in early RC do not take tumour location into account [1, 3, 4, 22, 23].

Based on the considerations above, the aim of this study was to investigate tumour location as a potential risk factor of LNM and recurrence in early RC, adjusting for potential confounding factors.

## Materials and methods

### Study design

This is a retrospective multicentre national cohort study on prospectively collected data. STROBE 2021 and RECORD 2021 guidelines were followed.

### Swedish colorectal cancer registry

All data were prespecified and retrieved from the Swedish colorectal cancer registry (SCRCR), a prospectively maintained national quality registry on colorectal cancer. The registry contains data on preoperative staging, perioperative surgical details, postoperative histopathology, oncologic treatment, and 5-year follow-up. The SCRCR has a high validity and coverage (99%) compared to the mandatory Swedish Cancer Registry [24]. Swedish guidelines recommend routine follow-up after surgical resection of non-metastatic colorectal cancer comprising clinical examination, CEA, CT scan of abdomen and thorax (1 and 3 years after surgery) as well as endoscopy (endoscopic control of colorectal anastomosis at 1 year, colonoscopy at 3 years).

### Study population

All patients with non-synchronous, non-metachronous T1 and T2 RC registered between January 2009 and March 2021 were retrieved from the SCRCR. The primary outcome measures were LNM as well as recurrence, and two corresponding cohorts were identified. Early RC was defined as pT1 and pT2, regardless of clinical or pathological N and M stage. The following exclusion criteria were applied to the LNM cohort; local resection, emergency surgery, inconsistent surgical technique, undetermined N stage, neo-adjuvant treatment, undetermined tumour location as well as missing data on any of the aforementioned variables. Additional exclusion criteria were applied to the recurrence cohort comprising distant metastasis or unknown distant metastasis status at diagnosis, death within one year of follow-up and patients lost to follow-up.

### Tumour location

Tumour location was defined as distance between anal verge and distal tumour border, measured in cm preoperatively by rigid rectoscopy. Tumour location was categorized as distal 0–5 cm, mid 5–10 cm and proximal 10–16 cm.

### Tumour location and lymph node metastases

Nodal status was categorized as either pN0 or pN+, the latter comprising both pN1 and pN2. The following factors were included as covariates, investigating impact of tumour location and LNM; age at diagnosis, sex, T stage, lymphovascular invasion (LVI), perineural invasion (PNI), mucinous subtype and histologic grade. LVI was defined as presence of lymphatic and/or vascular invasion. PNI was defined as invasion or contact with nerve, exceeding 33% of the nerve circumference. Mucinous subtype was defined as presence of extracellular mucin occupying more than 50% of the tumour volume. Histologic grade was classified as low- or high-grade cancer according to the Vienna classification and WHO guidelines [25, 26].

### Tumour location and cancer recurrence

To compare risk of recurrence according to tumour location, recurrence-free interval (RFI) and hazard ratios (HR) were analysed and adjusted with the following covariates; age at diagnosis, sex, T stage, N stage, lateral resection margin, LVI, PNI, mucinous subtype, lateral resection margin and histologic grade. Lateral resection margin was dichotomized with a threshold value of ≤ 1 mm, according to ESMO guidelines [27]. Recurrence was defined as both local and distant recurrence. RFI was defined as time from surgery to date of detected recurrence and patients were censored at date of most recent follow-up or death. The time cut-off was set to 6 years to account for a delay in the routine 5-year follow-up appointment.

### Statistical analyses

Chi-square, Fisher’s exact and Kruskal–Wallis rank sum test were used to compare categorical and numerical variables, respectively, in baseline characteristics. Uni- and multivariate logistic regression analyses were used to investigate the relationship between tumour location and LNM. Uni- and multivariate Cox regression analyses were used to determine the adjusted impact of tumour location on recurrence. Previously mentioned covariates were included in multivariable logistic and Cox regression analyses to adjust for influence on respective outcome. Kaplan–Meier curves, log–log plots and Schoenfeld residuals test were used for assuming proportional hazards. Missing values were imputed prior to logistic and Cox regression analyses using multiple imputation. Sensitivity analysis was performed by comparing analyses on complete cases and imputed data. Data are presented as median and interquartile range and *P* values < 0.05 were considered significant. Statistical analyses were performed using R software version 4.3.0 (R Core Team (2023). R: A Language and Environment for Statistical Computing. R Foundation for Statistical Computing, Vienna, Austria. URL https://www.R-project.org/) with the packages Survival [28] and mice [29].

### Ethical considerations

This study was approved by the Swedish ethical review authority (2020–06676) prior to study start. The study was carried out in accordance with the declaration of Helsinki. All data retrieved from SCRCR were coded and anonymity was guaranteed.

## Results

### Study population and tumour location

**Fig. 1:**
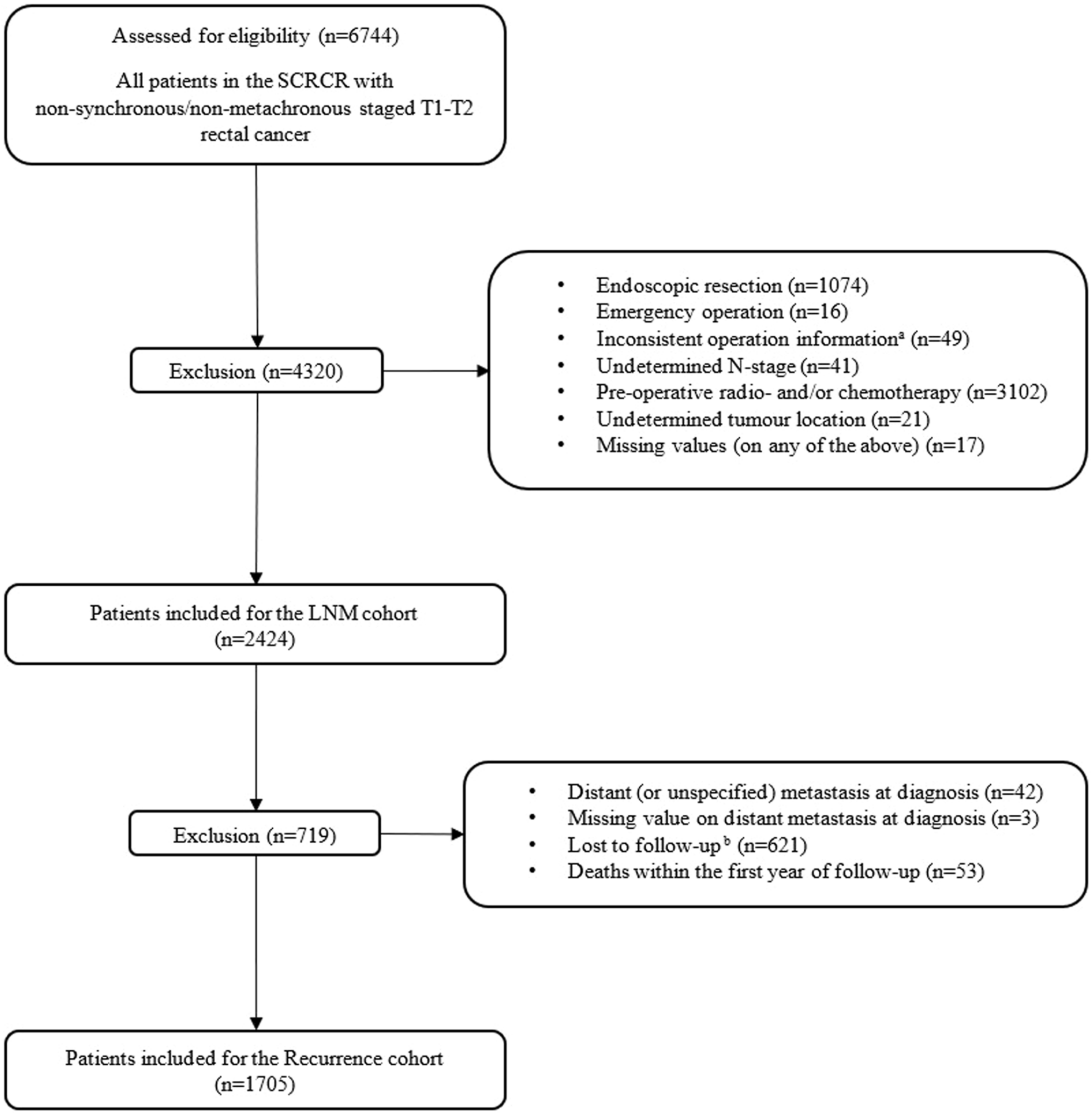
Inclusion and exclusion flowchart (^a^ right hemicolectomy (*n* = 2), left hemicolectomy (*n* = 3), unspecified colectomy (*n* = 22), laparotomy without resection (*n* = 1), other procedure (*n* = 21); ^b^ Missing value for recurrence or death (*n* = 618), missing follow-up or recurrence date (*n* = 3))

A total of 6744 patients with T1–T2 RC were assessed for eligibility, whereof 4320 were excluded and 2424 remained for analyses (Fig. 1). Median age at diagnosis was 71 years (64–78), 1361 (56.1%) were male and 736 (30.4%) had T1 tumours. Most tumours were located in mid (46.2%) and proximal rectum (38.8%). Patients with proximal tumours were younger compared to patient with distal RC (P value < 0.05) (Table 1). Distribution of tumour stage (T1/T2) and sex was comparable for distal, mid and proximal tumours (Table 1). The incidence of LVI, PNI, high histologic grade and mucinous subtype was higher in distal compared to mid and proximal tumours, significant for PNI (P value < 0.05) and mucinous subtype (P value < 0.001) but not for LVI (P value = 0.055) and histologic grade (P value = 0.266) (Table 1).

**Table 1:** Clinical and histopathological characteristics according to tumour location, LNM cohort

	Tumour location			Total
	Distal	Mid	Proximal	
Included patients	363 (15.0%)	1 121 (46.2%)	940 (38.8%)	2 424
Age at diagnosis^a^	73 (65–79)	71 (63–78)	70 (63–77)	71 (64–78)
Sex				
Female	160 (44.1%)	475 (42.4%)	428 (45.5%)	1 063 (43.9%)
Male	203 (55.9%)	646 (57.6%)	512 (54.5%)	1 361 (56.1%)
T stage				
pT1	103 (28.4%)	356 (31.8%)	277 (29.5%)	736 (30.4%)
pT2	260 (71.6%)	765 (68.2%)	663 (70.5%)	1 688 (69.6%)
Lymphovascular invasion				
Absent	284 (78.2%)	920 (82.1%)	790 (84.0%)	1 994 (82.3%)
Present	66 (18.2%)	160 (14.3%)	123 (13.1%)	349 (14.4%)
Missing	13 (3.6%)	41 (3.7%)	27 (2.9%)	81 (3.3%)
Perineural invasion				
Absent	315 (86.8%)	1031 (92.0%)	869 (92.4%)	2 215 (91.4%)
Present	24 (6.6%)	38 (3.4%)	28 (3.0%)	90 (3.7%)
Missing	24 (6.6%)	52 (4.6%)	43 (4.6%)	119 (4.9%)
Mucinous subtype				
Absent	314 (86.5%)	1017 (90.7%)	862 (91.7%)	2 193 (90.5%)
Present	36 (9.9%)	57 (5.1%)	45 (4.8%)	138 (5.7%)
Missing	13 (3.6%)	47 (4.2%)	33 (3.5%)	93 (3.8%)
Histologic grade				
Low-grade	316 (87.1%)	988 (88.1%)	843 (89.7%)	2 147 (88.6%)
High-grade	34 (9.4%)	90 (8.0%)	64 (6.8%)	188 (7.8%)
Missing	13 (3.6%)	43 (3.8%)	33 (3.5%)	89 (3.7%)
**Lymph node metastasis**	**66 (18.2%)**	**194 (17.3%)**	**203 (21.6%)**	**463 (19.1%)**
Harvested lymph nodes^a^	16 (12–22)	17 (13–23)	17 (13–24)	17 (13–23)
Missing	5 (1.4%)	13 (1.6%)	4 (0.4%)	22 (0.9%)

^a^Expressed as median and interquartile range

### Tumour location and LNM

In total, 463 (19.1%) of the 2424 included patients had LNM (Table 1). Median lymph node yield was 16 (12–22), 17 (13–23) and 17 (13–24) for distal, mid and proximal tumours, respectively (Table 1). Incidence of LNM was higher in proximal (21.6%) compared to mid (17.3%) and distal (18.2%) tumours, but the difference was not statistically significant in univariate logistic regression (Odds Ratio (OR) 1.239, Confidence Interval (CI) 0.910–1.688, P value = 0.173) (Table 2). When adjusting for covariates in multivariate logistic regression, proximal tumour location was found to be significantly associated with LNM (OR 1.504, CI 1.079–2.097, P value < 0.05) (Table 2). Amongst the included covariates, young age, T2 stage, LVI, PNI and high histologic grade were also found to be independent risk factors of LNM, with LVI being the predominant factor (Table 2).

**Table 2:** Uni- and multivariate logistic regression on tumour location and lymph node metastases, adjusting for potential covariates, LNM cohort

	Univariate analysis			Multivariate analysis		
	OR^a^	95% CI^b^	*p* value	OR	95% CI	*p* value
Tumour location						
Distal	1	Ref	Ref	1	Ref	Ref
Mid	0.942	0.692–1.282	0.703	1.090	0.783–1.516	0.610
Proximal	1.239	0.910–1.688	0.173	1.504	1.079–2.097	< 0.05
Age at diagnosis^c^	0.990	0.981–0.999	< 0.05	0.988	0.978–0.997	< 0.05
Sex						
Female	1	Ref	Ref	1	Ref	Ref
Male	1.153	0.939–1.416	0.175	1.109	0.892–1.378	0.352
T stage						
T1	1	Ref	Ref	1	Ref	Ref
T2	1.941	1.517–2.483	< 0.001	1.835	1.418–2.374	< 0.001
Lymphovascular invasion						
Absent	1	Ref	Ref	1	Ref	Ref
Present	4.713	3.694–6.013	< 0.001	4.132	3.200–5.336	< 0.001
Perineural invasion						
Absent	1	Ref	Ref	1	Ref	Ref
Present	3.870	2.529–5.922	< 0.001	2.246	1.389–3.632	< 0.001
Mucinous subtype						
Absent	1	Ref	Ref	1	Ref	Ref
Present	1.614	1.092–2.386	< 0.05	1.291	0.836–1.994	0.250
Histologic grade						
Low-grade	1	Ref	Ref	1	Ref	Ref
High-grade	2.117	1.529–2.931	< 0.001	1.515	1.046–2.194	< 0.05

^a^Odds Ratio; ^b^Confidence interval; ^c^OR per increasing year of age at diagnosis

### Tumour location and recurrence

**Fig. 2:**
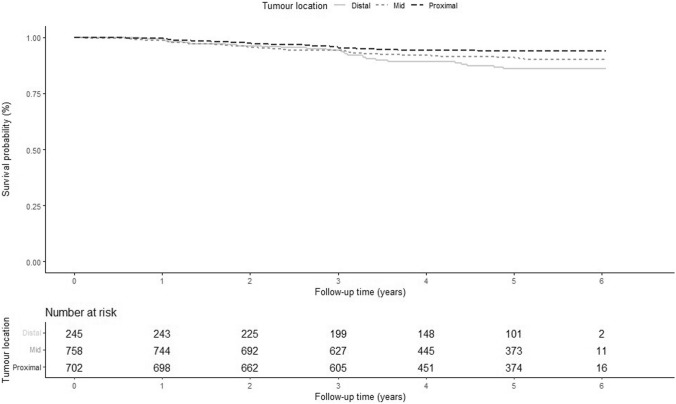
Kaplan–Meier curve, recurrence-free interval for distal, mid and proximal early rectal cancer

After applying additional exclusion criteria to the LNM cohort, 1705 patients remained for recurrence analyses (Fig. 1). Clinical and histopathological characteristics for this cohort are presented in Table 3. Distal tumours had a statistically significant lower lateral resection margin compared to mid and proximal tumours (P value < 0.05). In total, 130 (7.6%) patients were diagnosed with a recurrence during the 60 months median follow-up period and 5-year RFI was 92%. The majority of recurrences were distant (78.5%) and median time to recurrence was 25 (15–37) months. Incidence of recurrence was higher in distal (11.4%) compared to mid (8.3%) and proximal (5.6%) tumours and 5-year RFI was 86%, 91% and 94% for distal, mid and proximal tumours, respectively. In fact, both mid and distal tumour location were significant in both uni- and multivariate Cox regression analyses (mid: HR 1.592, CI 1.061–2.388, P < 0.05; distal: HR 2.051, CI 1.248–3.371, P < 0.05) (Table 4 and Fig. 2). Incidence of recurrences was comparable for distal tumours undergoing abdominoperineal resection 25/216 (11.6%), anterior resection 2/20 (10.0%) and Hartman’s procedure 1/9 (11.0%). Patients were referred for adjuvant therapy in 31/245 (12.7%), 90/758 (11.9%) and 100/702 (14.2%) for distal, mid and proximal tumours, respectively. Amongst included covariates, male sex, T2 stage, LNM and LVI were independently associated with recurrence, whereas lateral resection margin, PNI, mucinous subtype and histologic grade were not significantly associated with recurrence in multivariate analysis (Table 4).

**Table 3:** Clinical and histopathological characteristics according to tumour location, Recurrence cohort

	Tumour location			Total
	Distal	Mid	Proximal	
Included patients	245 (14.4%)	758 (44.4%)	702 (41.2%)	1 705
Age^a^	72 (64–79)	71 (63–78)	69 (63–76)	70 (63–77)
Sex				
Female	114 (46.5%)	327 (43.1%)	339 (48.3%)	780 (45.7%)
Male	131 (53.5%)	431 (56.9%)	363 (51.7%)	925 (54.3%)
T stage				
pT1	73 (29.8%)	245 (32.3%)	218 (31.1%)	536 (31.4%)
pT2	172 (71.2%)	513 (67.7%)	484 (68.9%)	1 169 (68.6%)
N stage				
pN0	202 (82.4%)	629 (83.0%)	562 (80.1%)	1 393 (81.7%)
pN +	43 (17.6%)	129 (17.0%)	140 (19.9%)	312 (18.3%)
Lateral margin (mm) ^a^	8 (4–13)	12 (7–19)	16 (10–25)	13 (8–20)
Missing	36 (14.7%)	81 (10.6%)	99 (14.1%)	216 (12.7%)
Distal margin (mm) ^a^	35 (20–46)	24 (15–40)	40 (25–55)	30 (20–50)
Missing	53 (21.6%)	99 (13.1%)	91 (13.0%)	243 (14.3%)
Lymphovascular invasion				
Absent	195 (79.6%)	624 (82.3%)	596 (84.9%)	1 415 (83.0%)
Present	39 (15.9%)	102 (13.5%)	83 (11.8%)	224 (13.1%)
Missing	11 (4.5%)	32 (4.2%)	23 (3.3%)	66 (3.9%)
Perineural invasion				
Absent	212 (86.5%)	694 (91.6%)	646 (92.0%)	1 552 (91.0%)
Present	12 (4.9%)	20 (2.6%)	19 (2.7%)	51 (3.0%)
Missing	21 (8.6%)	44 (5.8%)	37 (5.3%)	102 (6.0%)
Mucinous subtype				
Absent	213 (86.9%)	679 (89.6%)	645 (91.9%)	1 537 (90.2%)
Present	23 (9.4%)	43 (5.7%)	33 (4.7%)	99 (5.8%)
Missing	9 (3.7%)	36 (4.7%)	24 (3.4%)	69 (4.0%)
Histologic grade				
Low-grade	209 (85.3%)	665 (87.7%)	622 (88.6%)	1 496 (87.7%)
High-grade	26 (10.6%)	61 (8.0%)	51 (7.3%)	138 (8.1%)
Missing	10 (4.1%)	32 (4.2%)	29 (4.1%)	71 (4.2%)
Surgical resection				
APR^b^	216 (88.2%)	121 (16.0%)	23 (3.3%)	360 (21.1%)
Anterior resection	20 (8.2%)	526 (69.4%)	599 (85.3%)	1145 (67.2%)
Hartman’s operation	9 (3,6%)	111 (14.6%)	71 (10.1%)	191 (11.2%)
Sigmoid resection	0 (0%)	0 (0%)	9 (1.3%)	9 (0.5%)
Adjuvant therapy^c^				
No	212 (86.5%)	661 (87.2%)	596 (84.9%)	1469 (86.2%)
Yes	31 (12,7%)	90 (11.9%)	100 (14.2%)	221 (12.9%)
Missing	2 (0.8%)	7 (0.9%)	6 (0.8%)	15 (0.9%)
Recurrence	28 (11.4%)	63 (8.3%)	39 (5.6%)	130 (7.6%)

^a^Expressed as median and interquartile range, ^b^Abdominoperineal resection, ^c^Referred for adjuvant therapy

**Table 4:** Uni- and multivariate Cox regression analysis on tumour location and recurrence, adjusting for potential covariates, Recurrence cohort

		Univariate analysis			Multivariate analysis		
	Recurrence rate^a^	HR^b^	95% CI^c^	*p* value	HR	CI	*p* value
Tumour location							
Proximal	127	1	Ref	Ref	1	Ref	Ref
Mid	199	1.554	1.038–2.326	< 0.05	1.592	1.061–2.388	< 0.05
Distal	273	2.145	1.313–3.501	< 0.05	2.051	1.248–3.371	< 0.05
Age at diagnosis^d^		1.002	0.986–1.018	0.800	0.998	0.983–1.014	0.837
Sex							
Female	142	1	Ref	Ref	1	Ref	Ref
Male	211	1.481	1.034–2.123	< 0.05	1.467	1.020–2.111	< 0.05
T stage							
T1	99	1	Ref	Ref	1	Ref	Ref
T2	217	2.189	1.389–3.449	< 0.001	1.908	1.201–3.031	< 0.05
N stage							
N0	137	1	Ref	Ref	1	Ref	Ref
N +	378	2.751	1.920–3.942	< 0.001	2.127	1.447–3.127	< 0.001
Lateral resection margin							
> 1 mm	176	1	Ref	Ref	1	Ref	Ref
≤ 1 mm	384	2.482	0.908–6.781	0.076	1.927	0.683–5.437	0.213
Lymphovascular invasion							
Absent	149	1	Ref	Ref	1	Ref	Ref
Present	394	2.607	1.757–3.866	< 0.001	1.815	1.178–2.795	< 0.05
Perineural invasion							
Absent	170	1	Ref	Ref	1	Ref	Ref
Present	498	2.740	1.401–5.355	< 0.05	1.521	0.753–3.074	0.240
Mucinous subtype							
Absent	171	1	Ref	Ref	1	Ref	Ref
Present	321	1.823	1.021–3.256	< 0.05	1.351	0.718–2.542	0.348
Histologic grade							
Low-grade	170	1	Ref	Ref	1	Ref	Ref
High-grade	285	1.630	0.942–2.820	0.080	1.060	0.584–1.924	0.847

^a^Per 10,000 pat-years, based on complete data, ^b^Hazard ratio, ^c^Confidence interval, ^d^HR per increasing year of age at diagnosis

### Sensitivity analyses and missing data

Analyses performed on imputed data were compared with identical analyses performed on complete data (Tables S1 and Table S2). In LNM analyses, significance and ORs were similar comparing complete (n = 2172) and imputed (*n* = 2424) data, except for proximal tumour location (OR 1.358, CI 0.965–1.933, P value 0.084) and presence of high histologic grade (OR 1.445, CI 0.982–2.103, p value 0.058) not reaching significance in multivariate logistic regression on complete data. There was no difference in LNM rates between complete cases and cases with missing values (P value = 0.086). In recurrence analyses, significance and HRs were also similar comparing complete (*n* = 1250) and imputed (*n* = 1705) data, except mid tumour location not reaching significance in complete data (HR 1.528, CI 0.991–2.36, P value = 0.055). Lateral resection margin ≤ 1 mm was associated with recurrence in univariate analysis on complete data (HR 3.112, CI 1.147–8.452, P value < 0.05) but not on imputed data. There was no difference in survival between complete cases and cases with missing values (P value = 0.150).

## Discussion

Tumour location has become an increasingly important factor to consider in colorectal cancer related to a growing body of evidence suggesting different tumour biology as well as prognosis and oncologic outcome [11–13]. However, the importance and implication of tumour location on oncologic risk in early RC is poorly studied and remain to be determined. In the present retrospective multicentre national cohort study, we investigated tumour location as a potential predictor of LNM and recurrence in more than 2400 included patients with early RC. We found that distal and mid tumour location were significantly associated with risk of recurrence in uni- and multivariate Cox regression, with distal location being a predominant risk-factor of recurrence in early RC. In fact, patients with distal RC had twice as high risk of recurrence compared to patients with proximal tumours.

Herein, overall incidence of LNM in T1–T2 RC was 19.1% which is within the range of previous studies, reporting LNM in 10–24% for T1 and T2 RC [7–10, 30, 31]. Incidence of LNM differed slightly according to tumour location, with highest incidence in proximal (21.6%) compared to mid (17.3%) and distal (18.2%) RC, although this finding was not significant in univariate logistic regression. However, proximal tumour location was found to be a significant predictor of LNM in multivariate logistic regression. This discrepancy could possibly be explained by previously known risk factors of LNM, such as LVI and PNI, being more frequent in distal, compared to mid and proximal RC, counterbalancing the impact of proximal tumour location on incidence of LNM. Nevertheless, it is important to note that OR for proximal location was only 1.504 with a CI close to 1 (1.079–2.097) and the clinical implication of this finding is uncertain. In addition, proximal tumour location was not a significant risk factor of LNM in our sensitivity analyses comprising identical statistical computations on complete case data (*n* = 2172), as opposed to imputed data (*n* = 2424). This is most likely due to a type two error in the complete data-set in combination with the limited effect of proximal location on LNM as described above. Furthermore, tumour location and impact on LNM in early RC has scarcely been investigated in the literature and we managed to find only two comparable studies [7, 9]. Aytac et al. found that risk of LNM was higher in proximal, compared to distal RC, which is in line with our findings. In contrast, Nascimbeni et al., reported that distal RC was associated with an increased risk of LNM. However, that study included a limited number of patients with T1 RC (*n* = 119), which might explain this discrepancy. Notably, out of the covariates included in our analyses, LVI was the predominant risk factor of LNM with an OR of 4.132, even superseding the effect of T2 vs T1 (OR 1.835) on LNM risk. Thus, according to our results, the risk of LNM is higher in T1 LVI positive compared to T2 LVI negative patients. This finding is supported by numerous studies showing that LVI is a strong and independent predictor of LNM in RC [4, 6, 32]. In addition, PNI, high-grade cancer and low age were also found to be independent risk factors of LNM. PNI and high-grade cancer have previously been found to be associated with risk of LNM and high-grade cancer is included as one of the risk factors prompting subsequent surgical resection in T1 RC [1, 6]. However, PNI was present in merely 90 of 2424 cases (3.7%) hampering conclusions on its impact as a predictor of LNM in routine practice. Low age has also previously been proposed as an independent risk factor of LNM in early RC, supporting the notion that RC occurring at a younger age is of a more aggressive nature and more prone to metastasize [4, 31, 32].

To accurately predict risk of recurrence in early RC is important in order to guide both treatment and follow-up regimens. Herein, we found that 130 (7.6%) of the 1705 included T1–T2 patients were diagnosed with recurrences, during the 60-month (median) follow-up period. This finding is in line with several studies reporting that incidence of recurrence in T1–T2 RC ranges from 4.2% to 11.1% [33–37]. Importantly, we found that both mid and distal tumour location were significant risk factors of cancer recurrence in both uni- and multivariate Cox regression analysis. In fact, incidence of recurrence in patients with mid and distal RC were 8.6% and 11.4%, respectively, the latter being twice as high compared to patients with proximal tumours. Notably, distal tumour location and LNM had similar HR´s and thus similar negative impact on oncologic outcome, emphasizing the impact of distal tumour location on cancer recurrence. Indeed, several previous studies have reported worse prognosis for distal compared to mid and proximal RC, in support of our findings [14, 17]. In contrast, some studies have reported similar oncologic outcome regardless of location within the rectum [19, 20]. However, both of these studies differ from ours since they investigated overall survival and the study by Bhangu et al.[19] did not include tumour location in the multivariate analyses and the study by den Dulk et al. [20] excluded patients aged > 75. It is also important to note that the aforementioned studies included all stages of RC and very few studies have actually investigated impact of tumour location on oncologic outcome in early RC specifically. Nevertheless, two previous studies investigating recurrence in early RC, reported that distal tumour location was a risk factor for recurrence in uni- but not multivariate Cox regression [10, 35]. In this context, it is important to note that both of these studies are supportive of our findings, but they included less than 400 patients, respectively, and the lack of significance in multivariate analyses could possibly be explained by a type two error. Moreover, the increased risk of recurrence in distal compared to mid and proximal RC is not clearly understood. A possible explanation could be that tumour spread to lateral lymph nodes is gradually more frequent in mid and distal, compared to proximal RC, related to different lymphovascular anatomy [38]. Thus, it is possible that the increased risk of recurrence in mid and especially distal RC is a consequence of leaving lateral pelvic LNM untreated, when performing mesorectal excision, which does not routinely encompass lateral pelvic lymph node dissection [39]. Another explanation to the increased incidence of recurrence in distal tumours could be that surgeons compromise with resection margins to preserve bowel continuity and perform anterior resection on very low tumours. However, we found that incidence of recurrence was comparable for patients undergoing abdominoperineal resection, anterior resection and Hartman´s procedure for distal tumours, which is in line with a previous report [40]. In this context, it is important to stress that we have not compared surgical vs local resection in the present study and the increased risk of recurrence in distal early RC found herein, does not favour any specific resection method. Nevertheless, it is interesting to note that some previous studies have shown increased risk of recurrence after local compared to surgical resection of T1 RC [10, 41, 42], but they have not adjusted for tumour location, which is problematic in light of our present findings. In contrast, a previous study taking tumour location into account, comparing surgical and local resection of distal early RC reported similar oncologic outcome following both resection methods [43].

Among our included covariates, male gender, T2 stage, LNM and presence of LVI were also significant risk factors of recurrence. The association between LVI, LNM as well as higher T stage (T2 vs T1) and recurrence is not controversial and in line with the literature [17, 44, 45]. However, the increased risk of recurrence related to male sex is harder to explain. Interestingly, a previous study investigating differences in colorectal cancer related to sex, found that females have a survival benefit over males, lending some support to our finding [46].

Our study is strengthened by the large population-based sample size, prospectively collected data and multivariate analyses. Nevertheless, our study is limited by its retrospective design and for not being able to adjust for tumour budding, TME completion rate, MSI status and tumour size, since these parameters were not recorded in the registry for the entire study period. In addition, our data contain missing values, constituting another limitation. However, we performed imputation to account for missing data in our logistic and Cox-regression analyses and we performed sensitivity analyses.

In conclusion, risk of recurrence was found to be significantly associated with tumour location, with gradually increasing risk in mid and distal tumours, with twice as high risk of recurrence in distal compared to proximal tumours. Thus, our findings highlight the need for a higher awareness on the increased risk of poor oncologic outcome in mid and especially distal early RC, which might imply a more intense follow-up.

## Supplementary Information

Below is the link to the electronic supplementary material.

Supplementary file1 (DOCX 18 KB)

Supplementary file2 (DOCX 19 KB)
