# Supplementary material for: Is tumour location a dominant risk factor of recurrence in early rectal cancer?
Source: Surg Endosc. 2024 Dec 16;39(2):1056–66. doi: 10.1007/s00464-024-11413-6 (PMC11794355; doi:10.1007/s00464-024-11413-6)
Supplement: Supplementary file 1 — Supplementary file1 (DOCX 18 KB) [file 464_2024_11413_MOESM1_ESM.docx]

Table S1. Uni- and multivariate logistic regression on tumour location and lymph node metastases, adjusting for potential covariates, LNM cohort, complete case dataset (n=2172)

|  | Univariate analysis | | | Multivariate analysis | | |
| --- | --- | --- | --- | --- | --- | --- |
|  |  | | |  | | |
|  | OR ^a^ | CI ^b^ | p-value | OR | CI | p-value |
|  |  |  |  |  |  |  |
|  |  |  |  |  |  |  |
| Tumour location |  |  |  |  |  |  |
| Distal | 1 | Ref | Ref | 1 | Ref | Ref |
| Mid | 0.921 | 0.671-1.279 | 0.619 | 1.044 | 0.744-1.483 | 0.805 |
| Proximal | 1.162 | 0.845-1.615 | 0.362 | 1.358 | 0.965-1.933 | 0.084 |
|  |  |  |  |  |  |  |
| Age ^c^ | 0.990 | 0.981-0.999 | 0.036 | 0.988 | 0.977-0.998 | <0.05 |
|  |  |  |  |  |  |  |
| Sex |  |  |  |  |  |  |
| Women | 1 | Ref | Ref | 1 | Ref | Ref |
| Men | 1.123 | 0.909-1.393 | 0.291 | 1.069 | 0.852-1.343 | 0.566 |
|  |  |  |  |  |  |  |
| T stage |  |  |  |  |  |  |
| T1 | 1 | Ref | Ref | 1 | Ref | Ref |
| T2 | 1.764 | 1.369-2.293 | <0.001 | 1.628 | 1.248-2.141 | <0.001 |
|  |  |  |  |  |  |  |
| Lymphovascular invasion |  |  |  |  |  |  |
| Absent | 1 | Ref | Ref | 1 | Ref | Ref |
| Present | 4.990 | 3.879-6.419 | <0.001 | 4.370 | 3.359-5.683 | <0.001 |
|  |  |  |  |  |  |  |
| Perineural invasion |  |  |  |  |  |  |
| Absent | 1 | Ref | Ref | 1 | Ref | Ref |
| Present | 4.021 | 2.577-6.259 | <0.001 | 2.293 | 1.359-3.740 | <0.001 |
|  |  |  |  |  |  |  |
| Mucinous subtype |  |  |  |  |  |  |
| Absent | 1 | Ref | Ref | 1 | Ref | Ref |
| Present | 1.558 | 1.003-2.360 | <0.05 | 1.223 | 0.747-1.954 | 0.411 |
|  |  |  |  |  |  |  |
| Histologic grade |  |  |  |  |  |  |
| Low-grade | 1 | Ref | Ref | 1 | Ref | Ref |
| High-grade | 2.039 | 1.448-2.841 | <0.001 | 1.445 | 0.982-2.103 | 0.058 |
|  |  |  |  |  |  |  |

^a^ Odds Ratio; ^b^ Confidence interval; ^c^ OR per increasing year of age at diagnosis;
