# Supplementary material for: Is tumour location a dominant risk factor of recurrence in early rectal cancer?
Source: Surg Endosc. 2024 Dec 16;39(2):1056–66. doi: 10.1007/s00464-024-11413-6 (PMC11794355; doi:10.1007/s00464-024-11413-6)
Supplement: Supplementary file 2 — Supplementary file2 (DOCX 19 KB) [file 464_2024_11413_MOESM2_ESM.docx]

Table S2. Univariate and multivariate Cox regression analysis on tumour location and recurrence, adjusting for potential covariates, Recurrence cohort, complete case dataset(n=1362).

|  | Univariate Analysis | | | Multivariate analysis | | |
| --- | --- | --- | --- | --- | --- | --- |
|  |  | | |  | | |
|  | HR ^a^ | 95% CI ^b^ | *p*-value | HR | CI | p-value |
|  |  |  |  |  |  |  |
|  |  |  |  |  |  |  |
| Tumour location |  |  |  |  |  |  |
| Proximal | 1 | Ref | Ref | 1 | Ref | Ref |
| Mid | 1.477 | 0.960-2.271 | 0.076 | 1.528 | 0.991-2.36 | 0.055 |
| Distal | 2.057 | 1.203-3.518 | <0.05 | 1.865 | 1.082-3.21 | <0.05 |
|  |  |  |  |  |  |  |
| Age at diagnosis ^c^ | 1.001 | 0.984-1.018 | 0.894 | 0.999 | 0.983-1.016 | 0.916 |
|  |  |  |  |  |  |  |
| Sex |  |  |  |  |  |  |
| Women | 1 | Ref | Ref | 1 | Ref | Ref |
| Men | 1.492 | 1.008-2.207 | <0.05 | 1.516 | 1.020-2.254 | <0.05 |
|  |  |  |  |  |  |  |
| T stage |  |  |  |  |  |  |
| T1 | 1 | Ref | Ref | 1 | Ref | Ref |
| T2 | 2.294 | 1.33-3.955 | <0.05 | 2.076 | 1.199-3.597 | <0.05 |
|  |  |  |  |  |  |  |
| N stage |  |  |  |  |  |  |
| N0 | 1 | Ref | Ref | 1 | Ref | Ref |
| N+ | 2.801 | 1.910-4.106 | <0.001 | 2.162 | 1.435-3.256 | <0.001 |
|  |  |  |  |  |  |  |
| Lateral resection margin (mm) |  |  |  |  |  |  |
| > 1 | 1 | Ref | Ref | 1 | Ref | Ref |
| ≤ 1 | 3.113 | 1.147-8.452 | <0.05 | 2.631 | 0.951-7.276 | 0.062 |
|  |  |  |  |  |  |  |
| Lymphovascular invasion |  |  |  |  |  |  |
| Absent | 1 | Ref | Ref | 1 | Ref | Ref |
| Present | 2.634 | 1.737-3.994 | <0.001 | 1.915 | 1.213-3.025 | <0.05 |
|  |  |  |  |  |  |  |
| Perineural invasion |  |  |  |  |  |  |
| Absent | 1 | Ref | Ref | 1 | Ref | Ref |
| Present | 2.940 | 1.486-5.818 | <0.02 | 1.729 | 0.848-3.527 | 0.132 |
|  |  |  |  |  |  |  |
| Mucinous subtype |  |  |  |  |  |  |
| Absent | 1 | Ref | Ref | 1 | Ref | Ref |
| Present | 2.194 | 1.176-4.092 | <0.05 | 1.941 | 0.989-3.809 | 0.054 |
|  |  |  |  |  |  |  |
| Histologic grade |  |  |  |  |  |  |
| Low-grade | 1 | Ref | Ref | 1 | Ref | Ref |
| High-grade | 1.306 | 0.700-2.436 | 0.401 | 0.719 | 0.364-1.420 | 0.342 |
|  |  |  |  |  |  |  |

^a^ Hazard ratio; ^b^ Confidence interval; ^c^ HR per increasing year of age at diagnosis;
